# Supplementary material for: Citizen scientists and university students monitor noise pollution in cities and protected areas with smartphones
Source: PLoS One. 2020 Sep 11;15(9):e0236785. doi: 10.1371/journal.pone.0236785 (PMC7485857; doi:10.1371/journal.pone.0236785)

**S1 File. Instructions for noise monitoring application download and set up**

The iPhone app to download is called SPLnFFT, which you can find by searching in the App Store.

To set up SPLnFFT:

1. Go to the “Conf” page using the buttons in the middle of the screen

2. Scroll down to “Frequency analysis”

3. Where it says “Power SD or per band,” toggle the switch so that “3rdO” is highlighted in grey, instead of “PSD”

4. Scroll down to “Dosemetering”

5. Select “L50” (it should be highlighted in grey after you select it)

6. Every time you open the app: in the top left corner, under the “Pause” button, click on the word “FAST.” It should change to the “SLOW setting.

7. At the bottom of the screen, make sure your app is set to dB(A) (see below)

8. Click on the middle section of the screen until L50 appears (see below)


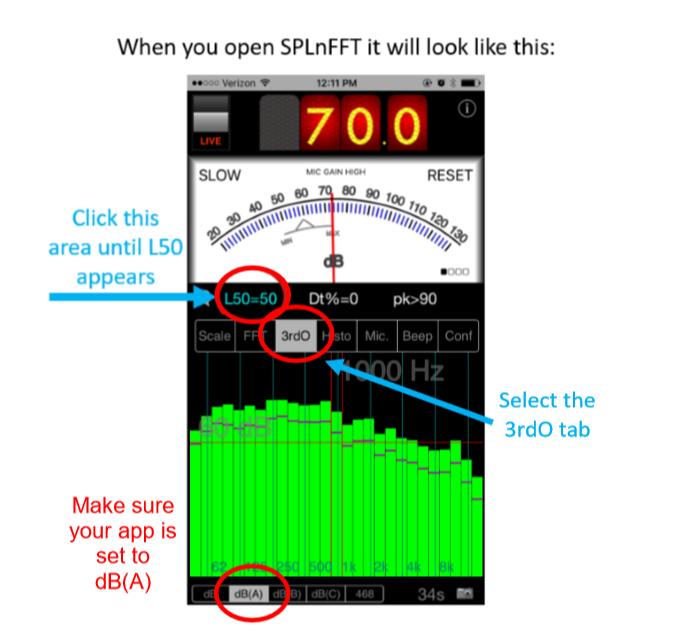

Supplement: S1 File — Instructions for citizen scientists to download and set up the SPLnFFT application prior to the noise monitoring event, can also be used as a handout. (DOCX) [file pone.0236785.s002.docx]
