# Supplementary material for: Citizen scientists and university students monitor noise pollution in cities and protected areas with smartphones
Source: PLoS One. 2020 Sep 11;15(9):e0236785. doi: 10.1371/journal.pone.0236785 (PMC7485857; doi:10.1371/journal.pone.0236785)
Supplement: S2 File — Instructions on calibrating the phone, and taking and sending a noise measurement for citizen scientists, can also be used as a handout. (DOCX) [file pone.0236785.s003.docx]

**S2 File. Instructions for citizen science noise monitoring in the field**

Once you have downloaded and set up the SPLnFFT app you are ready to calibrate and start recording noise! Please come to the event with your phone fully charged, but we do have external chargers available if you are running low on battery.

**Calibration:**

On the day of the event, organizers will help volunteers calibrate their phones so everyone’s measurements are standardized.

**Measurements:**

1. You will send each 20 second measurement via email to [(insert](mailto:soundmapBU@gmail.com) unique email address for event), we suggest adding it your phone as a contact at the start of the day.

2. Every 50 – 100 paces along your designated trail, pause and stand still to take a measurement.

3. When you stop to take a measurement, use the following guidelines to determine when you should start recording:

- Wait to begin the measurement until you do not hear any obvious airplane noise overhead, or the airplane noise is lower than background noise.
- If an airplane begins to fly overhead within the first 10 seconds of beginning the measurement, reset and begin the measurement again when the plane passes.
- If there is some special noise feature, such as a group of people walking along with a radio or yelling do not take the measurement.
- For places that have crowds of people treat the people as part of the background noise and take measurements.

4. When recording hold the iPhone face up, flat with the bottom pointing toward your body. Face your back to the wind.

5. Each recording will be 20 seconds long (if you go a few seconds over that’s ok!)

6. Note the **L50 value** (more details below), you will need to manually enter it. When entering the value **please only type the number**, do not type L50 = X

7. After each recording, send the measurement to (unique email address for event)

You’re ready to record!

*Please follow the iPhone instructions on the next page.* If you have any issues along the way, please text or call one of the researchers.


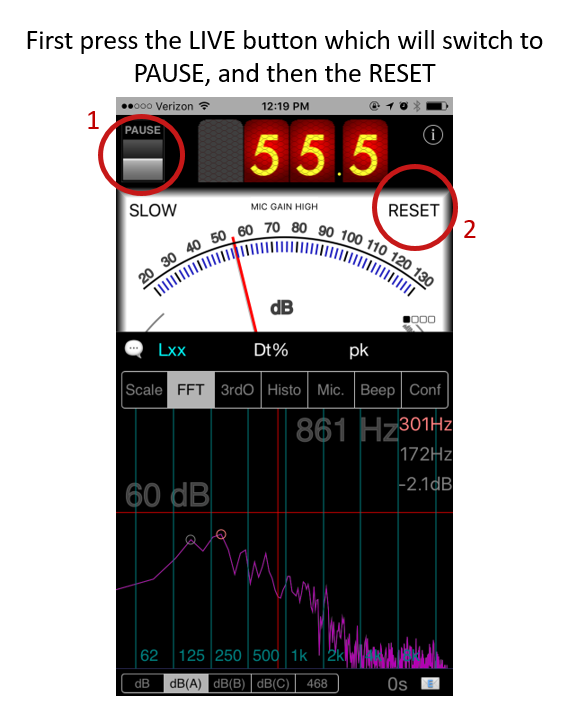


Frequently encountered problems:

1.
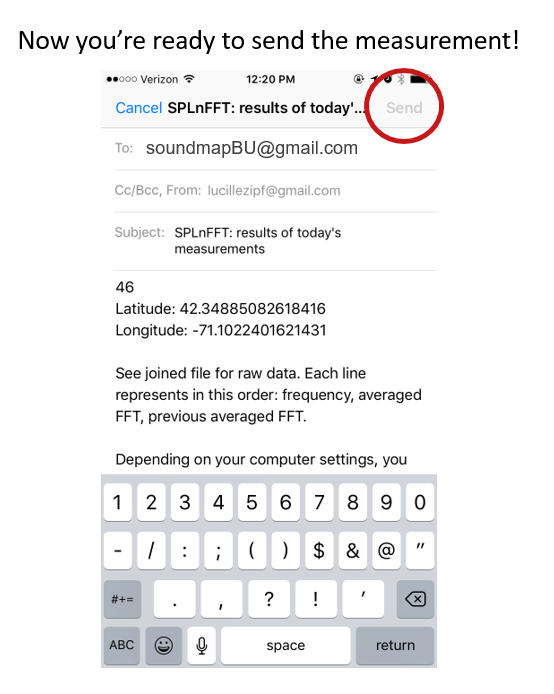
If you forget the L50 value while drafting the email, no sweat! Just press Cancel in the upper left hand corner of the email, and you will be returned to the paused SPLnFFT screen where you can check the L50 value again.
2. If the email feature appears to not be working, you may have not “Paused” the recording, check that you see “Pause” in the upper left hand corner of the app screen.
3. If it is taking a very long time to load the email screen (this should happen nearly instantly), check that you are in the 3rdO on the “Conf” page under “Frequency Analysis” – this should speed up the process.
4.
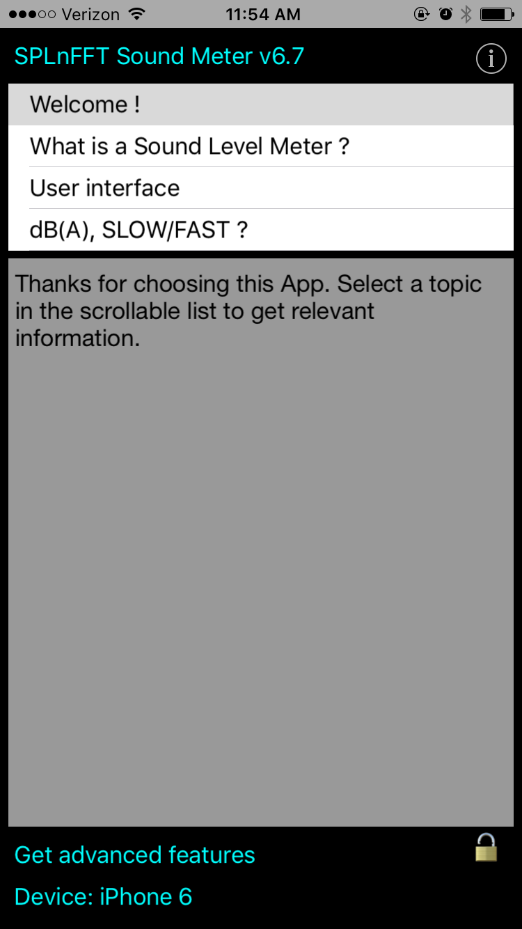
It is easy to accidentally change the setting to FAST in the upper right hand corner. So, every once in a while check that your app still reads SLOW under the “Pause” toggle.
5. SPLnFFT uses a lot of phone battery power, so please do not hesitate to request an external charger if you are running low.
6.
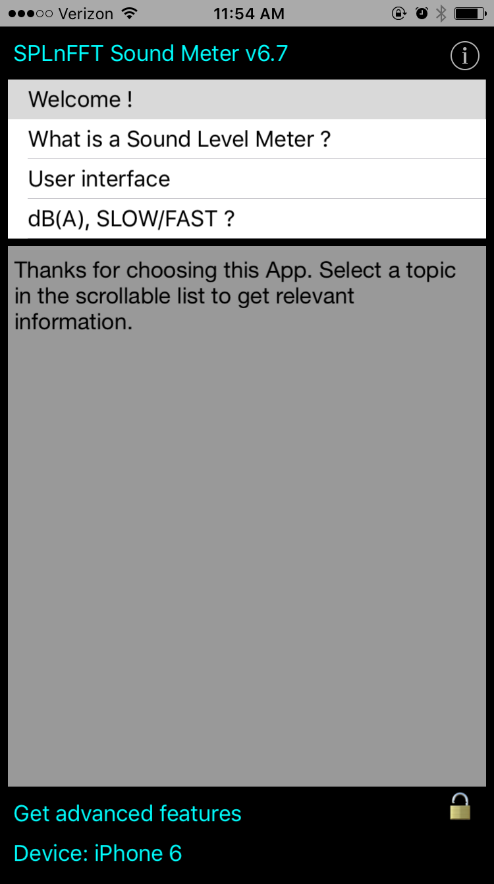
If the screen to the left appears in your app, you likely hit the information button, to switch back to the regular view hit the information button.
