# Supplementary material for: Citizen scientists and university students monitor noise pollution in cities and protected areas with smartphones
Source: PLoS One. 2020 Sep 11;15(9):e0236785. doi: 10.1371/journal.pone.0236785 (PMC7485857; doi:10.1371/journal.pone.0236785)
Supplement: S3 File — The survey questions that were sent to noise monitoring participants after a noise monitoring event. (DOCX) [file pone.0236785.s004.docx]

**S3 File. Post event survey**

**Post-event survey of citizen science volunteers**

1. How long did it take you to learn how to use the app, SPLnFFT?
2. 5-10 minutes
3. 10-20 minutes
4. 20+ minutes
5. Did you experience any problems taking and sending data? If yes, what problems?
   1. Yes
   2. No
      1. Comments:
6. Briefly, what did this project teach you about noise?
7. How likely are you to use this app in the future for another purpose? If likely, for what purpose?
   1. Will not use
   2. Unlikely
   3. Neutral
   4. Somewhat likely
   5. Very likely
      1. Comments:
8. What was your primary reason for participating in this citizen science project?
   1. Concern about noise pollution
   2. Learn more about noise pollution
   3. Help advance conservation science
   4. Enjoyment of being outdoors
   5. Support a good cause
