# Supplementary material for: Citizen scientists and university students monitor noise pollution in cities and protected areas with smartphones
Source: PLoS One. 2020 Sep 11;15(9):e0236785. doi: 10.1371/journal.pone.0236785 (PMC7485857; doi:10.1371/journal.pone.0236785)
Supplement: S4 File — A sample lesson plan for a laboratory activity that could be conducted using the described noise monitoring method. The learning outcomes students will achieve in this lab are to (1) understand the concept of noise pollution and tools used to measure noise levels, (2) apply this knowledge of noise monitoring with smartphones to create noise maps around their campus, and (3) evaluate the noise maps and draw conclusions about the local distribution and consequences of noise pollution. (DOCX) [file pone.0236785.s005.docx]

**S4 File. Lesson plan for noise monitoring laboratory experiment for high school or college courses**

Lab Title: Noise Mapping for Conservation Science

**Objectives**

The learning outcomes students will achieve in this lab are to (1) understand the concept of noise pollution and tools used to measure noise levels, (2) apply this knowledge of noise monitoring with smartphones to create noise maps around their campus, and (3) evaluate the noise maps and draw conclusions about the local distribution and consequences of noise pollution.

These outcomes can be achieved by introducing noise pollution to the class through a 10 to 20 -minute lecture on noise pollution, a 15-minute description of the noise monitoring methodology, and a one to two-hour lab exercise in which students take noise measurements of their campus. In the post-lab assignment, students create their own noise maps and describe the patterns of noise pollution that they have encountered. From these maps and observed patterns, students are then able to reach conclusions about noise sources on campus and surrounding areas, potential threats to human and wildlife health, and propose mitigation strategies.

**Introduction**

Noise pollution, or unwanted and disturbing sound, is a pervasive feature of modern life. Anthropogenic noise pollution from sources including road traffic, airplanes, and construction poses threats to the well-being of humans and wildlife, especially in populations around urban areas, where noise pollution tends to be greatest. Noise pollution is surprisingly common in protected areas, national parks, and rural areas, where it similarly affects both humans and wildlife. The impacts of noise pollution have been well-studied in the fields of public health and biology. For mammals, birds and other animals, noise pollution can interfere with communication, vigilance, and foraging, which in turn can negatively affect survival and reproduction. Human exposure to persistent noise can lead to cognitive impairment, distraction, stress, altered behavior, and ailments caused by these symptoms like insomnia, high blood pressure, and heart attacks; these can lead to increased mortality. By creating maps of noise in an area, or soundscapes, we can better understand the exposure of wildlife to noise pollution. Managers can then and use this information to inform conservation decisions aimed to mitigate the exposure of animals and people to excess noise.

To measure noise pollution, researchers and acoustical engineers commonly deploy sound recorders at one or a few locations and model noise levels at different distances from sound sources. This method, however, has serious limitations; noise pollution is extremely variable over time, and is strongly affected by local buildings, tree plantings, weather conditions, and topography. Models of soundscapes miss much of this variation, leading to inaccurate estimates of noise pollution problems in many areas.

In today’s lab we will overcome these limitations by taking advantage of recent advances in smartphone technology and use an existing research-grade smartphone app (SPLnFFT) to monitor sound along streets and in protected areas. We will then use OpenHeatMap.com (a free online mapping application) to create maps of noise pollution. After mapping your data, you will draw scientific conclusions and interpretations about the distribution and magnitude of noise pollution in our environment.

**Recommended Reading**

Buxton, R. T., McKenna, M. F., Mennitt, D., Fristrup, K., Crooks, K., Angeloni, L., & Wittemyer, G. (2017). Noise pollution is pervasive in U.S. protected areas. *Science*, *356*(6337), 531–533. http://doi.org/10.1126/science.aah4783

Francis, C. D., & Barber, J. R. (2013). A framework for understanding noise impacts on wildlife: An urgent conservation priority. *Frontiers in Ecology and the Environment*. http://doi.org/10.1890/120183

**Experimental Questions**

1. Will city streets be louder than green spaces?
2. Will sound intensity dissipate as you move from roads into protected areas?

**Materials & Set up**

***Table 1. Noise monitoring definitions.***

***_______________________________________________________________________________***

SPLnFFT: sound meter application for iPhone; includes location services (GPS), calibration features, and built-in mechanisms for data export via email and Dropbox

dBA: A weighted dB scale; emphasizes sound energy at frequencies where people and vertebrate animals have their most sensitive hearing thresholds

L50: median dBA over a sound recording; represents background noise levels and is not heavily skewed by brief, loud noises, such as car horns and bird calls

20 second measurement period: sound recording duration used in this study to capture the background noise of an environment

Instructions for SPLnFFT download, set up, and use available in the supplementary material.

We will monitor A-weighted L50 values in decibels during the lab, see the definitions above. Form groups of 2-4 students, one of whom must have an iPhone and the app SPLnFFT. The instructions for app download and setup are attached. Students will need to calibrate their phones using the in-app calibration feature.

Each group will then be assigned a route through the surrounding area, routes will follow city streets and cover local greenspaces.

**Procedure**

1. You will send each 20 second measurement via email to **(insert unique email made for class)**, we suggest adding it your phone as a contact at the start of the day.

2. Every 50 – 100 paces along your designated route, pause and stand still to take a measurement.

3. When you stop to take a measurement, use the following guidelines to determine when you should start recording:

- Wait to begin the measurement until you do not hear any obvious airplane noise overhead, or the airplane noise is lower than background noise.
- If an airplane begins to fly overhead within the first 10 seconds of beginning the measurement, reset and begin the measurement again when the plane passes.
- If there is some special noise feature, such as a group of people walking along with a radio or yelling do not take the measurement.
- For places that have crowds of people treat the people as part of the background noise and take measurements.

4. When recording hold the iPhone face up, flat with the bottom pointing toward your body. Face your back to the wind.

- Detailed instructions on how to take and send measurements are in the handouts attached

6. Note the **L50 value** (more details below), you will need to manually enter it. When entering the value **please only type the number**, do not type L50 = X

7. After each recording, send the measurement to **(insert unique email made for class)**

8. Once all measurements are sent your instructor will compile the class data and send it to the class in order to create a soundscape.

**Data Analysis**

Using the class data, each student is responsible for creating their own soundscape using the open-source online mapping software on openheatmap.com.

To create a map:

1. Create a file in Excel with three columns: L50, latitude and longitude
2. Save this file in the .csv (comma separated values) format
3. Go to openheatmap.com and follow instructions for data upload and map creation
4. Once your map is made, the website allows you to change the scale, which you should do if the scale does not span the range of L50 values in the class data set
5. Create your map! Soundscapes can be saved by exporting or screenshotting the finished product

Using your soundscape, answer the three questions posed above: Are city streets be louder than green spaces? Does sound intensity dissipate as you move from roads into protected areas?

**Questions to Consider**

- How do you think the noise affects humans and wildlife in the area?
- What are some management recommendations you would suggest to help conserve wildlife in the area?
