# Supplementary material for: Citizen scientists and university students monitor noise pollution in cities and protected areas with smartphones
Source: PLoS One. 2020 Sep 11;15(9):e0236785. doi: 10.1371/journal.pone.0236785 (PMC7485857; doi:10.1371/journal.pone.0236785)
Supplement: Attachment — Submitted filename: Review on PONE-D-19-23835.pdf [file pone.0236785.s006.pdf]

Review on manuscript number PONE-D-19-23835

titled

«Citizen scientists and university students monitor noise pollution in cities and protected areas with smartphones»

by Lucy Zipf, Richard B Primack, and Matthew Rothendler.

Summary and decision

This article reports on a single-day Citizen Science activity in which volunteers use smartphones to measure noise at different locations. Students process the single measurement data into a noise map. The topic of noise, noise pollution and noise protection is of great importance for society, animals and the environment. Occasions like this can help raise the citizens' awareness of noise and provide them with a scientific method to collect and interpret measurement data themselves. I recommend publishing this article when the following three main points of criticism can be addressed satisfactorily and the minor statements / questions are considered.

Major concerns

(M1) Concerning the theoretical background, it should be better worked out where the added value lies in the recording of one's own measurement data as a citizen. The keyword could be authentic data and ownership of data (see, for example, *Tara O'Neill and Angela Calabrese Barton: Uncovering Student Ownership in Science Learning: The Making of a Student Created Mini-Documentary* or *Enhag and Niedderer: Two dimensions of student ownership of learning during small-group work in physics and the references therein*).

(M2) A main characteristic of citizen science is that citizens learn to collect and interpret data independently. But in this study you control the data recording very strictly by specifying special calibration rules and even intervene people by telephone if you are sceptical about their measurement data. How is this compatible with the goal of making citizens as autonomous as possible and not degrading them to data producers?

(M3) The suitability to use smartphones in acoustics, also for measuring noise, has already been studied in depth in the physics education literature. It has been shown that they are reliable tools for educational purposes. Since sound measurement with smartphones is one key aspect to enable the CS project in this paper, this should not remain unmentioned.

Minor changes / Questions

In addition, I would like to see the following questions being answered and comments to be considered:

1. line 10: Noise distributions are also often simulated simply on the basis of theoretical models. This statement could be added.
2. line 14: in in
3. line 21 citicien science (2x)

4. line 46ff: Smartphones have also often been used in physics education (research) for learning in acoustics, e.g., see Hirth, Michael, Jochen Kuhn, and Andreas Müller. "Measurement of sound velocity made easy using harmonic resonant frequencies with everyday mobile technology." The Physics Teacher 53.2 (2015): 120-121. And Klein, P., Hirth, M., Gröber, S., Kuhn, J. & Müller, A. (2014). Classical experiments revisited: smartphones and tablet PCs as experimental tools in acoustics and optics. Physics Education, 49(4), 412.

   In particular, it was emphasized that the accuracy of measurement for educational purposes is sufficient.

5. line 49: please provide literature about using smartphones in the xontext of „air pollution“, I am not aware about that

6. line 57: create

7. line 66: so-called bioblitz

8. line 77: Please point out that the character of the »single-day event« is what adds the new value

9. line 80-81: It reads as if the combination of data sources would take place in this article. Please attenuate the statement.

10. Introduction in general: Please give more attention to the work of Shim et al 2016, which was published in the same journal. They took measurements over the period of one week, including working with Android devices. Please briefly present this work here.

11. Line 91: Here you write about a several-hour time period, elsewhere about a single-day event. Please stay consistent

12. Line 95-104: The mentioned buildings and areas could be highlighted in the pictures, possibly by a label.

13. Line 125-125: I have a few questions about the method: Why was the in-app calibration not used? How much do the results of an in-app calibration vary compared to a calibration with a standard instrument? Is it still possible to speak of citizen science if such a control is necessary? Does the calibration remain constant over the measuring period, i.e. over 90-120 minutes? What can you say about the measuring range of the app, especially for noises smaller than 30dB? Why did the citizens have to send the data after each measurement and why not send the complete data set at the end of the route?

14. Line 125: what is „the common standard“?

15. Line 134: Which criteria were used to select the routes and the density of the measuring points along the routes?

16. Line 160-162: That sounds like a lot of effort. Please comment on the purpose of citizen science. On the one hand you want to have very accurate measurement data and monitor it pretty closely, on the other hand citizens should have the responsibility for the measurement. How does that fit together?

17. Line 197: Wind can have a huge influence, as I know from my own measurements. Has it been checked that people have stood with their backs to the wind? Should a windscreen be used for the microphones in the future?

18. Line 218: What does local regression mean? What is local about it? What equation was used to fit the data and what is the message from the regression?

19. Line 238: Maybe I'm too pedantic there but "detailed" is a matter of definition. For a particle physicist, the amount of data you created is very small. Maybe you find a different description.
20. Line 244: what exactly is "our method". Please explain and summarize it briefly in the discussion
21. Line 255ff: Wouldn't it have been possible to ask the participants afterwards if they had used the smartphone again? The project took place about 2 years ago....
22. Line 263: You write that volunteers can monitor noise pollution independently. Doesn't that contradict the high level of control you have tried to achieve?
23. Line 294: Are there suitable apps for Android users that you can recommend and that have appeared in the meantime? Fig. 3 A: Why did you decide to make this division in the legend? It would be nice to be able to identify the areas mentioned in the text in the maps.
24. Additional References: Please also consider this work: Zappatore, Marco, Antonella Longo, and Mario A. Bochicchio. "Crowd-sensing our smart cities: A platform for noise monitoring and acoustic urban planning." (2017): 53-67.
