# Supplementary material for: Citizen scientists and university students monitor noise pollution in cities and protected areas with smartphones
Source: PLoS One. 2020 Sep 11;15(9):e0236785. doi: 10.1371/journal.pone.0236785 (PMC7485857; doi:10.1371/journal.pone.0236785)
Supplement: Attachment — Submitted filename: PONE-D-19-23835 review_an.pdf [file pone.0236785.s007.pdf]

manuscript assessment:
PONE-D-19-23835: "Citizen scientists and university students monitor noise pollution in cities and protected areas with smartphones"

COMMENTS
This is a very interesting contribution, and I liked the approach (citizen science + smartphones) very much. However, there are some minor and one major point to be improved; the latter is the reason to ask for a major (not minor) revision, but most probably is easy to address.

1) Major issue: lacking data and information in the "Materials & Methods" section
There is no information about technical data of the measurements you report, such as
- sensitivity
- accuracy
- precision
- spatial and temporal resolution

Please add these data, or at least estimates for them. Most importantly:
a) add a comparison measurement with a professional noise measurement equipment. As the data collection with your participant groups has already taken place, a measurement carried out by yourself would be sufficient.
b) add a comparison to a theoretical prediction for the decrease of road noise with distance (based on known result for a line/1D source)

Additionally, a discussion of the quality of data obtained in the "Discussion" section would be most useful for you readers.

2) Further Details

1) As you highlight two kinds of participant groups in the title, you should compare them (eg. by a chi^2 test) in terms of measurement quality, e.g. % removed (table 1) or other data you might have; for the former it looks as if there is no difference between citizen science participants and college students (of a health science track), and this is interesting for the community.

2) table 1: doesn't fit on the page

3) research for and refer to other lit. beyond noise monitoring in the spirit of your approach. eg:
a) earthquakes
Kong, Q., Allen, R. M., Schreier, L., & Kwon, Y. W. (2016). MyShake: A smartphone seismic network for earthquake early warning and beyond. *Science advances*, *2*(2), e1501055.
b) radioactivity
Laquai, B. Open Geiger citizen science project; Available online: www.opengeiger.de
Bottollier-Depois, J.F.; Allain, E.; Baumont, G.; Berthelot, N.; Clairand, I.; Couvez, C.; Darley, G.; Henry, B.; Jolivet, T.; Laroche, P.; et al. OPEN RADIATION: A collaborative project for radioactivity measurement in the environment by the public. EPJ Web Conf. 2017, 153, 08002. [CrossRef]
Keller, O., Benoit, M., Müller, A., & Schmeling, S. (2019). Smartphone and Tablet-Based Sensing of Environmental Radioactivity: Mobile Low-Cost Measurements for Monitoring, Citizen Science, and Educational Purposes. *Sensors*, *19*(19), 4264
Beser, A.M. How Citizen Science Changed the Way Fukushima Radiation is Reported. National Geographic Society Newsroom, 13 February 2016.
