# Supplementary material for: Citizen scientists and university students monitor noise pollution in cities and protected areas with smartphones
Source: PLoS One. 2020 Sep 11;15(9):e0236785. doi: 10.1371/journal.pone.0236785 (PMC7485857; doi:10.1371/journal.pone.0236785)
Supplement: Attachment — Submitted filename: Noise_response to reviewers_1July20.docx [file pone.0236785.s009.docx]

**Response to reviewers:**

**Major Suggestions from Reviewers:**

1. The spatial resolution values in table 1 are given with senseless precision (up to 5 digits and 2 decimals, while you know the area in km2 probably at best with an error of a few %). Please use a reasonable precision throughout the paper.

*Thanks for this suggestion. We fixed the issue and now report the values in whole numbers, which is much more appropriate precision.*
